# Resources for Enhancing Alzheimer’s Caregiver Health in Vietnam (REACH VN): study protocol for a cluster randomized controlled trial to test the efficacy of a family dementia caregiver intervention in Vietnam

**DOI:** 10.1186/s13063-022-06228-6

**Published:** 2022-05-09

**Authors:** Duyen Tran, Huong Nguyen, Thang Pham, Anh T. Nguyen, Hung T. Nguyen, Ngoc B. Nguyen, Bien H. Nguyen, Danielle Harvey, Laura Gitlin, Ladson Hinton

**Affiliations:** 1 Department of Neurology, University of California, Davis, Sacramento, CA USA; 2 School of Nursing, University of Minnesota, Minneapolis, MN USA; 3 National Geriatric Hospital, Hanoi, Vietnam; 4 Hanoi Medical University, Hanoi, Vietnam; 5 Hai Duong Provincial General Hospital, Hai Duong, Vietnam; 6 Department of Public Health Sciences, University of California, Davis, Davis, CA USA; 7 College of Nursing and Health Professions, Drexel University, Philadelphia, PA USA; 8 Department of Psychiatry and Behavioral Sciences, University of California, Davis, 2230 Stockton Blvd, Sacramento, CA 95817 USA

**Keywords:** Vietnam, Alzheimer’s, Dementia, Non-pharmacological caregiving intervention, Family caregiving, Global health

## Abstract

**Background:**

Alzheimer’s disease and related dementias (AD/ADRD) are a public health challenge for Vietnam because of its rapidly aging population. However, very few community-based programs exist to support people living with AD/ADRD and their family caregivers. Resources for Enhancing Alzheimer’s Caregiver Health in Vietnam (REACH VN) is a culturally adapted family caregiver intervention shown in a pilot study to be feasible and promising in terms of preliminary efficacy. We describe the protocol for a larger cluster randomized controlled trial (RCT) to test the efficacy of REACH VN among family caregivers of people living with dementia in a semi-rural area outside of Hanoi, Vietnam.

**Methods:**

Thirty-two clusters with approximately 350 caregivers will be randomized to either REACH VN intervention or enhanced usual care. REACH VN is a multicomponent intervention delivered in-home or by phone over the course of 2 to 3 months. To be eligible, family caregivers need to be ≥18 years old, be the person who provides the most day-to-day care for people living with dementia, and have a score ≥ 6 on the Zarit Burden Interview-4. The primary outcomes are caregiver burden (Zarit Burden Interview-12) and psychological distress (Patient Health Questionnaire-4). Secondary outcomes include caregiver somatic symptoms (Patient Health Questionnaire-15) and perceived stress (Perceived Stress Scale-10). These outcomes will be assessed at baseline, 3 months, and 6 months. Exploratory analyses to examine potential mediators of primary outcomes are also planned.

**Discussion:**

To our knowledge, this is the first large-scale study to test the efficacy of a community-based family dementia caregiver intervention in Vietnam. Results from this study will help inform efforts to widely deliver the REACH VN intervention or similar community-based family dementia caregiver support programs in Vietnam and other low- and middle-income countries (LMIC).

**Trial registration:**

ClinicalTrials.govNCT04542317. Registered on 9 September 2020

## Administrative information

Note: the numbers in curly brackets in this protocol refer to SPIRIT checklist item numbers. The order of the items has been modified to group similar items (see http://www.equator-network.org/reporting-guidelines/spirit-2013-statement-defining-standard-protocol-items-for-clinical-trials/).

**Table:** 

Title {1}	Resources for Enhancing Alzheimer's Caregiver Health in Vietnam (REACH VN): study protocol for a cluster randomized controlled trial to test the efficacy of a family dementia caregiver intervention in Vietnam
Trial registration {2a and 2b}	The study was registered on clinicaltrials.gov on September 9, 2020 (NCT04542317).
Protocol version {3}	March 3, 2022.
Funding {4}	The study is supported by National Institute on Aging (NIA) under award number R01AG064688 (Hinton and Nguyen MPI) and the National Center for Advancing Translational Sciences (NCATS), National Institutes of Health (NIH), through grant UL1 TR001860 for use of REDCap.
Author details {5a}	Duyen Tran^1^, B.S., Huong Nguyen^2^, Ph.D., Thang Pham^3,4^, M.D., Ph.D., Anh T. Nguyen^3,4^, M.D., Ph.D., Hung T. Nguyen^3,4^, M.D., Ph.D., Ngoc B. Nguyen^3^, M.D., Ph.D., Bien H. Nguyen^5^, M.D., Danielle Harvey^6^, Ph.D., Laura Gitlin^7^, Ph.D., Ladson Hinton^8*^, M.D. ^1^Department of Neurology, University of California, Davis, Sacramento, CA, USA ^2^School of Nursing, University of Minnesota, Minneapolis, MN, USA ^3^National Geriatric Hospital, Hanoi, Vietnam ^4^Hanoi Medical University, Hanoi, Vietnam ^5^Hai Duong Provincial General Hospital, Hai Duong, Vietnam ^6^Department of Public Health Sciences, University of California, Davis, Davis, CA, USA ^7^College of Nursing and Health Professions, Drexel University, Philadelphia, PA, USA ^8^Department of Psychiatry and Behavioral Sciences, University of California, Davis, Sacramento, CA, USA *Correspondence Ladson Hinton Department of Psychiatry and Behavioral Sciences, University of California, Davis 2230 Stockton Blvd, Sacramento, CA 95817, USA.Email: lwhinton@ucdavis.edu
Name and contact information for the trial sponsor {5b}	National Institutes of Health (NIH) 9000 Rockville Pike, Bethesda, MD 20892, USA
Role of sponsor {5c}	The NIH plays no role in the study design, collection, management, analysis, interpretation of data, or writing and publication of the final manuscript.

## Introduction

### Background and rationale {6a}

Alzheimer’s disease and related dementias (AD/ADRD) are among the most disabling and costly neurodegenerative brain diseases [1]. Over the next 30 years, low- and middle-income countries (LMIC) such as Vietnam are undergoing a dramatic demographic transition that will substantially increase the number of older adults, including those with AD/ADRD. It is anticipated that in 2030, 63% of the people with AD/ADRD will live in LMIC, and this proportion anticipates increasing to 71% in 2050 [2]. In LMIC where formal support services are scarce, people living with AD/ADRD are cared for by family members who often sacrifice time and income to provide care [3]. In Vietnam, family members have traditionally taken care of older adults [4, 5], and this is reinforced by the Law on the Elderly [6].

As one of the fastest-aging countries [7], Vietnam has not well-prepared to provide effective care and support to people living with AD/ADRD and their family caregivers. Due to day-to-day care and lack of supports, AD/ADRD adversely impacts the psychological and physical morbidity, social isolation, and financial hardship on family caregivers [8]. For instance, Vietnamese dementia caregivers report increased in financial hardship, physical health problems (e.g., sleep disturbance and fatigue), and psychological distress (e.g., anxiety and depression) [5, 9, 10].

Although AD/ADRD are not curable, a large and growing base of evidence exists in high-income countries (HIC) for psychosocial interventions to improve caregiver outcomes [11, 12]. Culturally tailored dementia caregiver interventions have been demonstrated to reduce caregiver burden and depression, increase Alzheimer’s disease (AD) knowledge and self-efficacy in utilizing support services, and have positive effects on well-being of Vietnamese dementia caregivers in the United States (US) [13–15].

While evidence-based non-pharmacological treatments exist in HIC, the efficacy of these interventions has not been tested in Vietnam and many other LMICs. Multicomponent interventions, which often include psychoeducation, caregiver stress reduction, caregiver skill-building and coping, pleasant event scheduling, support groups, and information about community services, are a promising approach to improve outcomes (e.g., depression, burden, quality of life) for AD/ADRD family caregivers in Asia [16]. Also, multicomponent interventions provide disease education and skills training that are tailored to the specific concerns and care preferences of caregivers. Given their tailoring feature and person-family centric approaches, multicomponent interventions may be adaptable to countries and cultures that differ from the populations for which they were initially designed and tested. One of the most widely disseminated and tested models, Resources for Enhancing Alzheimer’s Caregiver Health (REACH), is effective in multicultural populations in the US [11, 17–19] and has been successfully adapted for use in Hong Kong [20]. An evidence-based intervention, Resources for Enhancing All Caregivers Health in the Department of Veterans Affairs (REACH VA) [17, 21], was adapted in Vietnam and tested in a pilot cluster randomized controlled trial (RCT) of 60 family caregivers [22], showing strong evidence of feasibility and preliminary efficacy. In this pilot study, caregivers who received the intervention showed significantly lower caregiver burden and psychological distress levels than those in the control group [23]. This study builds upon our pilot study to test the efficacy of Resources for Enhancing Alzheimer’s Caregiver Health in Vietnam (REACH VN) in a larger cluster RCT embedded in a community setting outside Hanoi using a Phase III clinical trial design [24].

### Objectives {7}

#### Primary objectives

The primary objectives of this study are to measure changes in caregiver burden and psychological distress (primary outcomes) between baseline (T0) and 3 (T1) and 6 (T2) months follow-up and to compare these outcomes in the two groups (i.e., REACH VN and enhanced control). We will test the hypothesis that family caregivers who receive the intervention will show lower caregiver burden and psychological distress over time compared with those in the control group.

#### Secondary objectives

The secondary objectives of this study are to measure changes in caregiver somatic symptoms and perceived stress (secondary outcomes) between baseline (T0) and 3 (T1) and 6 (T2) months follow-up and to compare outcomes in the two groups (i.e., REACH VN and enhanced control).

#### Exploratory analyses

This study will also have exploratory analyses to examine mechanisms. We will examine if changes in primary outcomes are mediated by caregiver self-efficacy or dementia knowledge gain or both.

### Trial design {8}

**Fig. 1:**
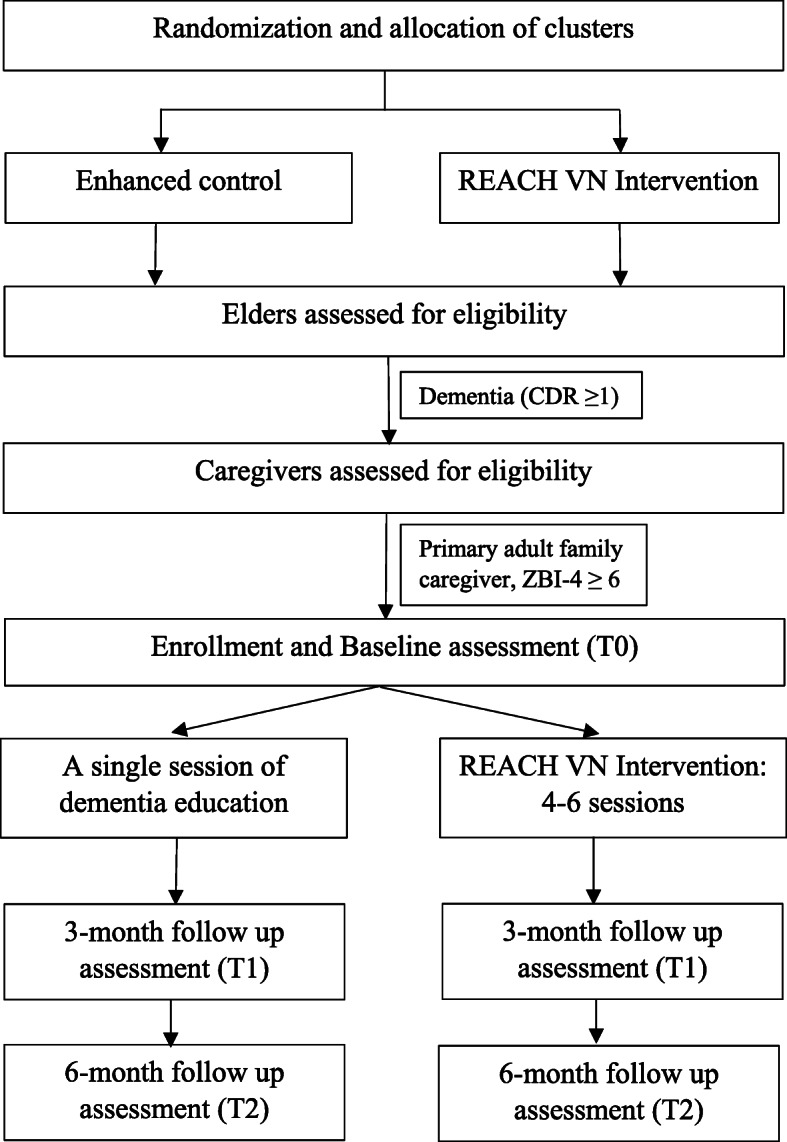
CONSORT flow diagram of REACH VN

**Fig. 2:**
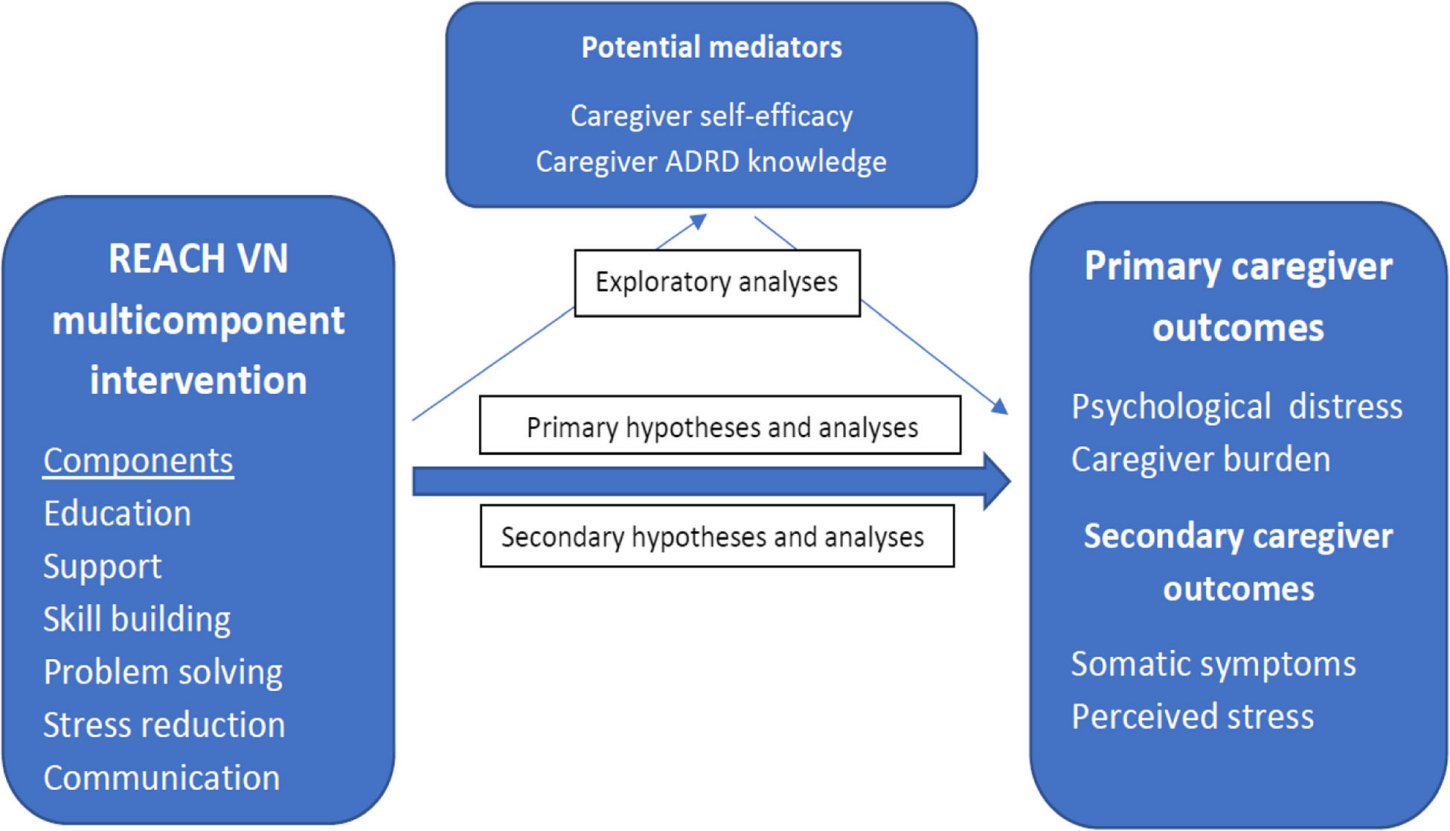
REACH VN impacts on caregiver outcomes and potential intervention mechanisms

This study is a cluster randomized controlled trial (RCT) with two arms. Clusters (i.e., communes) will be randomized with a ratio 1:1 for two groups: intervention (*n* = approximately 16 clusters or 175 caregivers) or enhanced usual care (*n* = approximately 16 clusters or 175 caregivers) (Figs. 1 and 2).

## Methods: participants, interventions, and outcomes

### Study setting {9}

The study will be conducted in two districts (Thanh Mien District and Gia Loc District) within Hai Duong, a province in Northern Vietnam. This site was chosen for several reasons, including a longstanding relationship and history of collaboration, including research, with the National Geriatric Hospital (NGH). Second, the proximity to the NGH (approximately a one-hour drive) will make face-to-face training and supervising possible to complement videoconferencing. Third, Hai Duong offers a blend of rural and semi-urban areas that roughly approximates Vietnam itself and enhances generalizability of the findings; more than two-thirds of Vietnam’s population reside in rural areas. This will allow us to examine rural versus semi-urban conditions as potential moderators of intervention effects at the cluster level.

### Eligibility criteria {10}

#### Cluster inclusion criteria

To be eligible for the study, clusters will need to be in Hai Duong and have a minimum of 5 participants and a maximum of 15 participants.

#### Individual participant inclusion criteria

To be eligible to participate, a family member will need to be the identified adult (i.e., age 18 and above) who is the primary informal (i.e., unpaid family member) caregiver (i.e., the person who provides the most time day-to-day care) to an older adult with dementia living in the community. If the primary caregiver is not available to participate, an alternate family member who provides substantial care (i.e., at least 4 h/day) to an older adult with dementia will be eligible to participate. In addition, caregivers will need to score ≥ 6 on the Zarit Burden Interview-4 (ZBI-4) [25, 26]. All participants will need to live in designated clusters (i.e., communes) in Hai Duong, Vietnam.

#### Individual participant exclusion criteria

The study will exclude caregivers who are unable to consent, individuals who are not yet adults (e.g., infants, children, teenagers), and prisoners.

### Who will take informed consent? {26a}

Informed consent will be obtained by study research staff who are researchers in Vietnam, have fluency in the language/dialects, and have completed local human subject research training. Because of the minimal risk nature of this behavioral intervention, verbal consent will be obtained.

### Additional consent provisions for collection and use of participant data and biological specimens {26b}

Because no ancillary studies are planned and no biological specimens will be collected, additional consent provisions are not applicable.

### Interventions

#### Explanation for the choice of comparators {6b}

In this study, the comparator will be clusters in which participants receive a single educational session about dementia including written materials at the time of enrollment. The decision to offer an educational session as an enhanced control condition is based on ethical considerations. Given the lack of basic services and information for people living with dementia in Vietnam, we decided that it was important to offer basic education on the condition as part of the control group. This is consistent with the approach taken in our pilot study which demonstrated benefits of enhanced control in terms of improvements in primary outcomes [23]. This is also consistent with how REACH II was conducted—enhanced care but it involved 3 education type sessions [27].

#### Intervention description {11a}

REACH VN is a culturally adapted version of REACH VA in the US and is a multicomponent intervention that includes risk assessment, education, support, skill-building, problem-solving, cognitive restructuring, stress management, and communication [23]. It is highly structured and manualized and includes materials for caregivers, including a notebook with practical tips and strategies on how to deal with difficult aspects of caregiving. It is suitable for delivery by staff who do not have a high level of professional training [17, 21].

In our pilot study, when we adapted the REACH VA intervention in Vietnam, we made numerous modifications in terms of changing to content, context/delivery, and training interventionists to make it culturally appropriate and suitable for the context of a semi-rural area in Vietnam. In the intervention manual and caregiver notebook, we made changes in scripts, examples, and resources appropriate to the culture and literacy level of the target population and increased the amount of time devoted to AD education. Moreover, we engaged the male head of the household in the initial session to facilitate participation and retention and multiple family members to participate in the intervention when appropriate. In addition to the standard REACH VA training, we applied principles of Buddhism to the training to enhance interventionist skills and conducted a small case-series to give interventionists hands-on experience [23].

Family caregivers in the REACH VN intervention group will participate in an enrollment visit and then receive 4 to 6 additional one-hour sessions over the course of 2–3 months. The visits will occur every 1–2 weeks depending on the needs and availability of family caregivers. The first visit will occur within 2 weeks of caregiver screening. The intervention will be delivered by research staff (e.g., nurse, social worker, or community health worker) who have been trained and certified to deliver the intervention by staff at the NGH. The sessions will be delivered in the home (or another place of the participant’s choice) or by telephone.

Two interventionist staff will participate in each visit, with one interventionist designated as the primary person conducting the intervention and the second person playing a supportive role during the visit. Fidelity will be assessed in two ways. At the beginning of each intervention session, interventionists will take a picture at participants’ home (e.g., take a selfie at the gate, take a picture of the gate, or take a picture of an object) and send it to their supervisor team. After their supervisors confirm that they received the picture, interventionists will immediately delete it. At the end of each visit, interventionists will complete a standardized treatment form (i.e., Interventionist Delivery Assessment (IA)) that summarizes the visit. The IA forms will be given to the Hai Duong Project Manager and to NGH. At the end of the active phase, caregivers are provided contact information of interventionists. During the maintenance phase from the closure session till the 6-month assessments, caregivers are encouraged to reach out to interventionists by telephone if they face caregiving problems. Interventionists also actively reach out to caregivers monthly for about 15 min for a wellness check in with caregivers. All maintenance sessions are documented. Supervision of all interventionists will take place during a weekly supervision meeting (in-person or by phone) with staff at the NGH.

#### Criteria for discontinuing or modifying allocated interventions {11b}

As part of the consent process, participants are informed that this is a voluntary study which they can leave at any time for any reason. If they withdraw from the study, they will be given the option of having their records destroyed.

#### Strategies to improve adherence to interventions {11c}

Our study will use several methods to improve adherence and fidelity to the intervention, focusing on aspects related to participants, interventionists, and outcome assessors.

##### Participant adherence

To enhance participant adherence to the intervention and decrease the risk of dropout, a participant will be matched with a designated interventionist who will serve as their contact person to remind them of intervention sessions and assessment appointments and answer questions and concerns that they may have related to the study.

##### Interventionist adherence

To enhance interventionist adherence, our intervention is fully manualized with detailed instruction for each session. All interventionists will receive training from members of the research team who are certified and experienced in the intervention delivery. All interventionists will complete a standardized treatment form after each visit (e.g., documentation of key elements of delivery), and these forms will be reviewed by supervisors and discussed at weekly supervision meetings.

##### Data collection adherence

To reduce outcome biases and enhance outcome assessor adherence, all assessments will be conducted by an independent institution, the Institute of Population Health and Development (PHAD). All outcome assessors will go through training sessions to enhance measurement reliability. After the training, each outcome assessor will complete at least one mock session which will be evaluated by their supervisors. Once assessors are certified by their supervisors, they will collect data with study participants. PHAD will perform data completion checks weekly to minimize missing data. Assessments will be conducted face-to-face or by phone.

#### Relevant concomitant care permitted or prohibited during the trial {11d}

Family caregivers and people living with dementia can receive routine care through the commune health station and, as necessary, at the provincial hospital. Because of the dearth of community supports for caregivers of people living with dementia, we do not anticipate any concomitant care related to the focus of this study.

#### Provisions for post-trial care {30}

This is a minimal risk study, so no post-trial care is planned.

### Outcomes {12}

Outcomes will be assessed by research staff from PHAD who are masked to allocation. Participants in both groups will be assessed at baseline (T0), 3 months (T1), and 6 months (T2).

#### Primary outcome measures

Primary outcomes are caregiver psychological distress as measured by the Patient Health Questionnaire-4 (PHQ-4) [28] and caregiver burden as measured by the Zarit Burden Interview-12 (ZBI-12) [25].

The Patient Health Questionnaire-4 items (PHQ-4) will be used to measure psychological distress, including 2 items from PHQ-2 to measure signs of depression and 2 items from GAD-2 to measure anxiety. Caregivers will be asked to rate on a scale of 0 (not at all) to 3 (nearly every day) about how often they have been bothered by the following problems over the last 2 weeks: feeling nervous, anxious, or on edge; not being able to stop or control worrying; feeling down, depressed or hopeless; and little interest or pleasure in doing things. The total score ranges from 0 to 12, with higher scores indicating greater levels of psychological distress [28].

The Zarit Burden Interview-12 items (ZBI-12) will be used to measure caregivers’ levels of burden. For each item, caregivers will be asked to rate on a scale of 0 (never) to 4 (nearly always). The total score ranges from 0 to 48, with higher scores indicating more burden [25].

#### Secondary outcome measures

Secondary outcomes will include change in stress level as measured by the Perceived Stress Scale-10 (PSS-10) [29] and change in somatic symptoms as measured by the Patient Health Questionnaire-15 (PHQ-15) [30].

The Perceived Stress Scale-10 items (PSS-10) will be used to measure caregivers’ stress levels. Caregivers will be asked how often they felt a certain way on a scale of 0 (never) to 4 (very often) within the past month. The total score ranges from 0 to 40, with higher scores indicating higher levels of perceived stress [29].

The Patient Health Questionnaire-15 items (PHQ-15) will be used to assess somatic symptoms. Caregivers will be asked to rate on a 3-point scale (0 = not bothered at all, 1 = bothered a little, 2 = bothered a lot) for how much they have been bothered by each of the 15 somatic symptoms (e.g., stomach pain, headaches, shortness of breath) during the past 7 days. The total score ranges from 0 to 30, with higher scores indicating greater severity of somatic symptoms [30].

#### Exploratory analyses

We will assess two different potential mediators of the intervention effect (see Fig. 2): caregiver self-efficacy as measured by the Caregiving Self-Efficacy Scale [31] and dementia knowledge assessed by the Dementia Knowledge Scale [32].

The Caregiving Self-Efficacy Scale includes 15 items that will be used to assess caregivers’ ability and confidence in managing dementia, including self-efficacy for obtaining respite, responding to disruptive patient behaviors, and controlling upsetting thoughts about caregiving. For each item, caregivers will be asked to rate their degree of confidence from 0 to 100 where a 0% confidence means that they cannot do it at all and a 100% confidence means they are certain they can do it [31].

The Dementia Knowledge Scale is a 11-item true/false scale assessing caregivers’ knowledge of dementia, including general information, symptom, treatment, cause, and prognosis of dementia. The total score is calculated based on the number of correct responses (0–11), with higher scores indicating greater knowledge of dementia [32].

### Participant timeline {13}

**Fig. 3:**
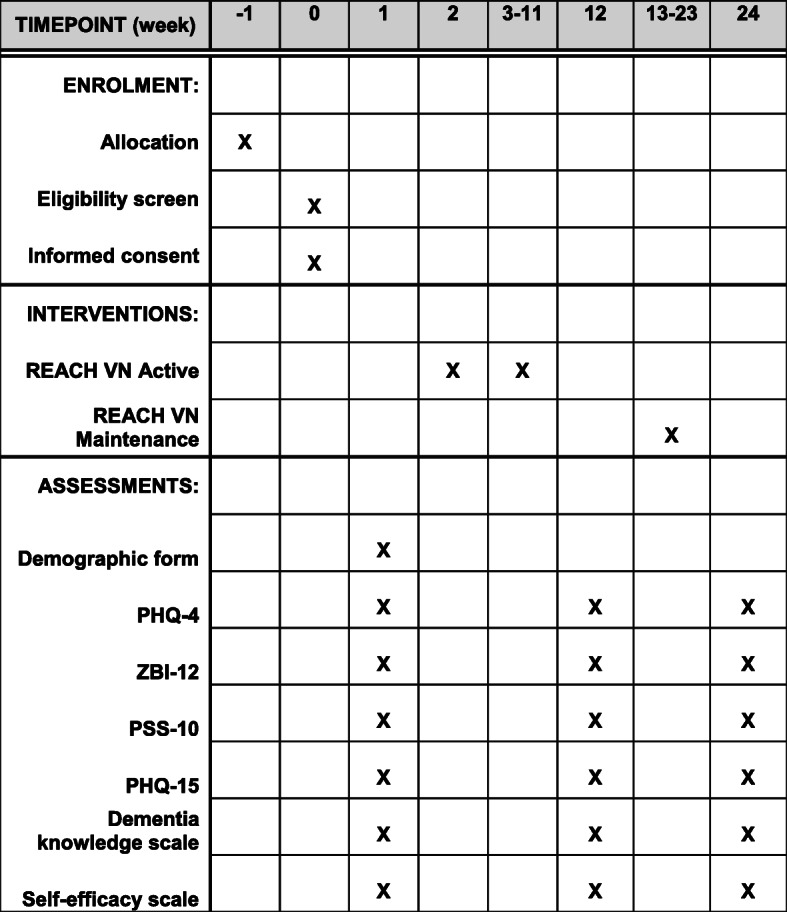
Participant timeline

See the participant timeline in Fig. 3.

### Sample size {14}

Our planned sample size is 32 clusters which have approximately 350 family caregivers. Assuming a two-sided test, alpha = 0.025 (to account for the two primary outcomes), and 32 clusters of approximately 11 participants each (*n* = 16 clusters or 175 caregivers in intervention and *n* = 16 clusters or 175 caregivers in control), we will have 80% power to detect a difference between groups of 0.46–0.57 standard deviations (SD) post-intervention if the intraclass correlation coefficient ranges from 0.1 to 0.2, as suggested by our pilot data [23]. These detectable effect sizes are approximately half of those observed in our pilot study, which allows for reduced effectiveness of intervention implementation at the local level.

### Recruitment {15}

Village health workers will first be trained by the NGH team about how to identify people “at risk” of dementia, using a short instrument developed by the NGH team. The instrument employed three simple questions on memory, language, and personality taken from the Assessment of Cognitive Complaints Toolkit-Alzheimer's Disease (ACCT-AD), combined with a checklist of likely symptoms on these three domains. Once trained, village health workers will then go through the list of all people aged 60 and above living in their village and mark anyone “at risk” of dementia, using the instrument above. For each “at risk” person, village health workers will also indicate their primary caregivers based on the village health workers’ knowledge of the family.

Based on the list of “at risk” people, commune health station staff will contact each person by phone or in-person to invite them for a clinical assessment at the health station or in the home, depending on their preference. In case the “at risk” person is unable to communicate (e.g., they are bed-ridden and unable to talk), commune health station staff will contact the family to make an invitation. When contacting the “at risk” person or their family, commune health station staff will also ask for the primary caregiver of the “at risk” person. They will invite both the “at risk” person and their primary caregiver to come for the clinical assessment. The assessment will be conducted by neurologists and psychiatrists from Hai Duong Provincial General Hospital and NGH, in collaboration with commune health station staff.

At the end of the clinical assessment, primary caregivers of older adults who meet criteria for dementia (i.e., Clinical Dementia Rating (CDR) ≥ 1) will be invited to participate in a brief screen to determine their eligibility for the RCT. If the primary caregiver is not present at the time of the clinical assessment, one of the members of the assessment team will reach out to the older adult’s home by phone or in-person to identify and screen the primary caregiver. Family caregivers who meet the inclusion criteria will be invited to participate in the study and verbal consent will be obtained.

## Assignment of interventions: allocation

### Sequence generation {16a}

We plan a stratified block randomization, stratified by rural (based on the Vietnamese government categorization) versus semi-urban status, so that groups are balanced across time and by rural versus semi-urban status. Communes will be the unit of randomization, and randomization assignments will be generated using a block randomization function in R for each stratum with a 1:1 ratio for two groups. Because the screening will be conducted in three phases over the course of 3 years, randomization will be done at three times prior to each phase of screening.

### Concealment mechanism {16b}

Randomization occurs at the cluster level, and there is no concealment at the individual level at the time of consent to the study.

### Implementation {16c}

The UC Davis Biostatistician will be responsible for generating randomization codes and assigning randomization codes to communes. The biostatistician will communicate cluster assignment to the principal investigator (PI) to convey to the study team in Vietnam of when communes have been randomized.

## Assignment of interventions: blinding

### Who will be blinded {17a}

Staff who conduct outcome assessments will be blinded to the allocation of family caregivers and cluster assignment.

### Procedure for unblinding if needed {17b}

Not applicable as participants are not blinded.

## Data collection and management

### Plans for assessment and collection of outcomes {18a}

Staff from PHAD will conduct assessments for study participants. All outcome assessments will be conducted face-to-face in the home or another place of the participants’ choosing. During COVID-19, assessments can be conducted via phone due to social distancing directives at local areas. The initial assessment will occur within 1 week of the enrollment visit and subsequent visits will occur within a 2-week window of time at 3 months and 6 months post-enrollment. Data entry will be conducted on tablets using Research Electronic Data Capture (REDCap) [33].

### Plans to promote participant retention and complete follow-up {18b}

Several strategies will be used to enhance retention. Participants in both the intervention group and the enhanced control group will be given the option of meeting interventionists in another setting, such as the local health station. We will also be flexible in arranging times to meet with participants, such as after work or on the weekend if these times work best for them. Participants will also be given the option of conducting some intervention sessions or outcome assessments by phone to enhance retention. Interventionists will make monthly calls for about 15 min each call to keep in-touch with participants in the intervention group.

### Data management {19}

To ensure firewalls between scientific leadership and data management/statistical investigators, all data entry and management will be handled by PHAD, an outside research institute in Vietnam that has experience in survey research. REDCap [33] will be used to collect outcomes data. All data will be stored on a secure server in Vietnam. The de-identified data will be transferred electronically through an encrypted zip file from the organization to the study biostatistician located at UC Davis. After the study, the key linking personal identifiers to the survey data will be destroyed.

### Confidentiality {27}

Procedures for maintenance and confidentiality include (1) assigning each participant a unique identifier; (2) data collected will be labeled using the unique identifier and stored separately from the key linking personal information (e.g., name, date of birth, address, phone number) and identifiers; (3) data will be kept on a secure server that is only accessible to research staff; (4) at the conclusion of the study, the key linking identifiers and personal information will be destroyed; and (5) all research personnel in the US who have access to the data will receive training on conducting human research (e.g., NIH online course) and all investigators and research staff in Vietnam will also participate in the local equivalent of this training.

### Plans for collection, laboratory evaluation, and storage of biological specimens for genetic or molecular analysis in this trial/future use {33}

Not applicable. No samples collected.

## Statistical methods

### Statistical methods for primary and secondary outcomes {20a}

Preliminary analyses will compare the groups at baseline on participant characteristics, clinical outcomes, and measures of mechanism (caregiving self-efficacy and dementia knowledge) using two-sample *t*-tests or Wilcoxon rank-sum tests for continuous measures and chi-squared tests or Fisher’s exact tests for categorical variables. If groups differ at baseline on any of these measures, they will be included as covariates in later models. Analyses will be similar for the primary (caregiver burden and psychological distress) and secondary outcomes (caregiver perceived stress and somatic symptoms). The primary assessment of intervention effects will be based on intent-to-treat analyses. Similar analyses will be conducted for the primary and secondary outcomes. Due to the cluster-randomized design of the RCT, data will be multi-level, with baseline and follow-up assessments on participants who are nested within communes. Multi-level mixed effects regression models will be used to assess the efficacy of the intervention, in which the 3- and 6-month assessments are used as outcomes and the baseline assessment is used as a covariate. These models are more flexible than standard repeated measures analysis of variance or analysis of covariance in that the form of the variance-covariance structure of the clustered data, both within-person and within-commune, can be explicitly modeled. The main factors of interest will be group and visit, along with their interaction, to assess differences between groups post-intervention as well as three months following completion of the intervention. These models will include participant- and commune-level random effects. If there is substantial variation in the timing of the 3- and 6-month assessments across participants (i.e., 3-month assessment happens closer to 2 months for some and closer to 4 months for others), we will use time since the baseline assessment as the time variable rather than the categorical visit number and evaluate group differences in change over time.

Model diagnostics will be performed for all models to test the underlying assumptions of the models and interactions between group and baseline measurement, transformations, non-linear models, or repeated measures approaches for non-normal data will be considered as needed.

### Interim analyses {21b}

Because this study was determined to be minimal risk by our Institutional Review Board (IRB) and based upon our preliminary studies and prior published studies, no interim analyses are planned.

### Methods for additional analyses (e.g., subgroup analyses) {20b}

Secondary analyses will evaluate the “as-treated” effect by using the number of attended intervention sessions, rather than group, as the predictor of interest, to assess whether those that participated in more of the intervention experienced greater change (those in the enhanced control group would have zero attended intervention sessions). Exploratory analyses will compare groups on caregiver self-efficacy and dementia knowledge post-intervention using similar models. These measures will then be incorporated into the models for the primary outcomes as additional independent variables to assess whether group differences in the primary outcomes are attenuated, an indication of possible mechanism for the intervention.

### Methods in analysis to handle protocol non-adherence and any statistical methods to handle missing data {20c}

The extent of missing data and patterns of missingness will be assessed once all study participants have completed the study. If needed, multiple imputation methods will be used and sensitivity analyses, including complete case analysis, and alternative approaches will be conducted to evaluate the impact of the imputation on study findings.

### Plans to give access to the full protocol, participant-level data, and statistical code {31c}

Clinical and outcomes data will be deposited at http://dataverse.harvard.edu, which is an NIH-funded repository. All relevant and referenced data will be deposited no later than the time of online publication date for the main trial outcome results. We will further abide by requirements set by the journal where we publish results regarding making the underlying data and statistical code available.

## Oversight and monitoring

### Composition of the coordinating centre and trial steering committee {5d}

For the duration of the trial, oversight and coordination of day-to-day intervention activities will be conducted locally through weekly meetings of interventionists and administrative staff from Hai Duong Provincial General Hospital together with senior interventionists and study investigators from the Vietnam National Geriatric Hospital. Additionally, a steering committee composed of senior investigators from the Vietnam National Geriatric Hospital and the two study principal investigators in the US (University of California, Davis and University of Minnesota) will meet biweekly throughout the trial to monitor overall trial progress, timeliness of assessments conducted by Institute for Population Health and Development, an independent organization, adverse events, screening activities, and other ethical issues. Screening activities are also coordinated locally by staff from the Vietnam National Geriatric Hospital, Hai Duong Provincial General Hospital, and local community health station staff, including village health workers. There is no trial steering committee or stakeholder and public involvement group.

### Composition of the data monitoring committee, its role, and reporting structure {21a}

This study will have an independent safety officer (SO), approved by the funding agency, who will meet 1–2 times each year to review the progress of the study, including adverse events, procedures for maintaining the confidentiality of data, and the quality of data collection, management, and analyses. If needed, the SO will also review any issues that arise in terms of conflict of interest or serious adverse events. The SO will report directly to the funder (i.e., NIA).

### Adverse event reporting and harms {22}

Adverse events (AE) will include untoward or unfavorable mental or physical health-related occurrences experienced by a participant that are temporally associated with participation in the research, regardless of the relatedness to the research. Examples of such events that might occur include incidents of brief hospitalization, caregiver distress or depression, instances of elder abuse or neglect, and other unanticipated events. Serious adverse events (SAE) will be further defined as those events that seriously jeopardize the participant’s health, including prolonged hospitalization, suicide attempts, and death. The overall goal of this plan is to have both serious adverse events (SAE) and non-serious adverse events (NSAE) that are viewed as likely (i.e., 50% or higher) related to study participation reported to both the UCD IRB and NIH in a timely fashion.

As a routine part of training, intervention study staff will be educated to monitor for these events and to report them to the research staff. Interventionists or other research staff will complete the AE Field Report Form and provide this to the Hai Duong project lead within 48 h. The project lead in Hai Duong will send all AE Field Report Forms to RCT leads at the NGH and the interventionist supervisors within 48 h. Upon receiving the report, RCT leads must confirm that they have received the report and are available to act upon it. RCT leads will review the AE Field Report Form, complete the AE Form, and if it is a SAE, complete the SAE Form and send to study PIs within 48 h. Within 2 working days of receipt of the information about the SAE, the PI will share this information with the research team and the SO. If the SAE is viewed as likely (i.e., > 50% probability) of being related to participation in the study, the SAE will be reported to the UCD IRB and to NIH within 2 working days of this determination with the goal of reporting all such events to UCD IRB and to the NIH Program Officer (PO) within 5 working days of the time the PI becomes aware of the event. NSAE will be shared with the core members of the research team at their regular biweekly meetings. If NSAE are deemed as possibly related to study participation, they will be reported to the SO within one week, and if the SO views them as “probably” (i.e., > 50% likelihood) of being related to study participation, they will be reported to the UCD IRB and NIH PO within 5 working days. All SAE and NSAE that are viewed as not likely to be related to research participation will be reported on an annual basis to the NIH PO as part of the progress report as well as to the UCD IRB.

### Frequency and plans for auditing trial conduct {23}

Throughout the trial period, an independent safety officer (SO), who has been approved by the funding agency (National Institutes of Health/National Institute of Aging), will meet 1–2 times each year to review the study protocol, monitor study progress, and review adverse events and other ethical issues or challenges. A report of these meetings will be submitted to the funding agency. Because the risk of this caregiving intervention is considered low, we will not have a data safety monitoring board for this trial.

### Plans for communicating important protocol amendments to relevant parties (e.g., trial participants, ethical committees) {25}

Any modifications to the protocol which may impact the conduct of the study, including changes of study objectives, study design, or study procedures will require a formal amendment to the protocol. Any modifications will be approved by IRB at UC Davis and NGH prior to implementation. Relevant changes will also be made to the study protocol in clinicaltrials.gov.

### Dissemination plans {31a}

Our project findings will be disseminated through publications and conferences (e.g., Alzheimer’s Association International Conference, National Dementia Conference in Vietnam) to help inform efforts to promote community-based family dementia caregiver support programs in Vietnam as well as other LMICs.

## Discussion

Our study will be the first one in Vietnam and among the first culturally adapted family caregiver intervention programs in LMIC in Asia to test the efficacy of a psychosocial intervention to support Alzheimer’s family caregivers. As a phase III trial, our cluster RCT will be implemented in a community setting with community providers but will retain aspects of an efficacy trial, including rigorous assessment of outcomes and fidelity monitoring. To prepare for full implementation of the RCT in a practice setting, after the start of the study, we will conduct a formative evaluation guided by the Reach, Effectiveness, Adoption, Implementation, and Maintenance (RE-AIM) framework to anticipate challenges and develop strategies and modifications to inform a next stage pragmatic effectiveness trial [34]. This study has several strengths, including being embedded in a local community and the involvement of frontline workers in the delivery of the intervention. If successful, the results of this study are anticipated to inform a future study to scale-up and disseminate this intervention more broadly in Vietnam. Supporting family caregivers of people living with dementia has been identified as a priority in the early stages of developing a national dementia plan for Vietnam [35]. The evidence generated by this study will be of high relevance to policy makers as Vietnam plans for its rapidly aging population. We believe that findings from this study can significantly influence dementia care practice and help Vietnam to develop support services for people living with dementia and their caregivers.

## Trial status

Recruiting started on October 15, 2020. The current protocol is version 1.1 of March 3, 2022. Recruitment is anticipated to be completed by March 30, 2024.

## Abbreviations

ACCT-AD: Assessment of Cognitive Complaints Toolkit-Alzheimer’s Disease
AD: Alzheimer’s disease
AD/ADRD: Alzheimer’s disease and related dementias
AE: Adverse event
CDR: Clinical Dementia Rating
HIC: High-income countries
IA: Interventionist Delivery Assessment
IRB: Institutional Review Board
LMIC: Low- and middle-income countries
NCATS: National Center for Advancing Translational Sciences
NGH: National Geriatric Hospital
NIA: National Institute on Aging
NIH: National Institute of Health
NSAE: Non-serious adverse events
PHAD: Institute of Population, Health and Development
PHQ-4: Patient Health Questionnaire-4
PHQ-15: Patient Health Questionnaire-15
PO: Program officer
PSS-10: Perceived Stress Scale-10
RCT: Randomized controlled trial
REACH VA: Resources for Enhancing All Caregivers Health in the Department of Veterans Affairs
REACH VN: Resources for Enhancing Alzheimer’s Caregiver Health in Vietnam
RE-AIM: Reach, Effectiveness, Adoption, Implementation, and Maintenance
REDCap: Research Electronic Data Capture
SAE: Serious adverse events
SO: Safety officer
UCD: University of California, Davis
ZBI-4: Zarit Burden Interview-4
ZBI-12: Zarit Burden Interview-12
